# Expression and role of fibroblast activation protein-alpha in microinvasive breast carcinoma

**DOI:** 10.1186/1746-1596-6-111

**Published:** 2011-11-08

**Authors:** Xing Hua, Lina Yu, Xiaoxiao Huang, Zexiao Liao, Qi Xian

**Affiliations:** 1 Department of Pathology, the Forth Affiliated Hospital of Jinan University, 510220 Guangzhou, China; 2 Department of Pathology, Guangzhou Red Cross Hospital, 510220 Guangzhou, China; 3 Department of Pathology, College of Basic Medicine, Southern Medical University, 510515 Guangzhou, China; 4 Department of Pathology, Nanfang Hospital, Southern Medical University, 510515 Guangzhou, China

**Keywords:** fibroblast activation protein-alpha, microinvasion breast carcinoma, diagnosis

## Abstract

**Background:**

Diagnosis of ductal carcinoma in situ (DCIS) in breast cancer cases is challenging for pathologist due to a variety of in situ patterns and artefacts, which could be misinterpreted as stromal invasion. Microinvasion is detected by the presence of cytologically malignant cells outside the confines of the basement membrane and myoepithelium. When malignant cells invade the stroma, there is tissue remodeling induced by perturbed stromal-epithelial interactions. Carcinoma-associated fibroblasts (CAFs) are main cells in the microenvironment of the remodeled tumor-host interface. They are characterized by the expression of the specific fibroblast activation protein-alpha (FAP-α), and differ from that of normal fibroblasts exhibiting an immunophenotype of CD34. We hypothesized that staining for FAP-α may be helpful in determining whether DCIS has microinvasion.

**Methods:**

349 excised breast specimens were immunostained for smooth muscle actin SMA, CD34, FAP-α, and Calponin. Study material was divided into 5 groups: group 1: normal mammary tissues of healthy women after plastic surgery; group 2: usual ductal hyperplasia (UDH); group 3: DCIS without microinvasion on H & E stain; group 4: DCIS with microinvasion on H & E stain (DCIS-MI), and group 5: invasive ductal carcinoma (IDC). A comparative evaluation of the four immunostains was conducted.

**Results:**

Our results demonstrated that using FAP-α and Calponin adjunctively improved the sensitivity of pathological diagnosis of DCIS-MI by 11.29%, whereas the adjunctive use of FAP-α and Calponin improved the sensitivity of pathological diagnosis of DCIS by 13.6%.

**Conclusions:**

This study provides the first evidence that immunostaining with FAP-α and Calponin can serve as a novel marker for pathologically diagnosing whether DCIS has microinvasion.

## Background

With widespread use of mammographic screening, many cases of breast cancer are now detected at an early stage. This has led to an increased incidence of not only in situ but also microinvasive carcinoma. Today, ductal carcinoma in situ (DCIS) accounts for 25% to 30% of breast cancer cases that are detected in the population-screening programs [1,2]. In contrast, DCIS with microinvasion (DCIS-MI) is an uncommon pathologic entity that represents < 1% of breast cancers [2,3].

In the histological examination of DCIS-MI, the main objective is to identify invasive focus or foci, because the therapy for patients with pure in situ carcinoma differs from that of patients with in situ carcinoma associated with microinvasive breast cancer [4]. Microinvasion is detected with the presence of cytologically malignant cells outside the confines of the basement membrane and myoepithelium [5]. Histological evaluation of minuscule foci of microinvasion is often difficult for the pathologist, because a variety of in situ patterns and artefacts could be misinterpreted as stromal invasion [6-9]. However, during the carcinogenesis of DCIS-MI, there is tissue remodeling induced by the perturbed stromal-epithelial interactions. Stromal fibroblasts in the microenvironment of the remodeled tumor-host interface are known as carcinoma-associated fibroblasts (CAFs) [9]. They are the main cells in the stroma and are characterized by the expression of the specific fibroblast activation protein-alpha (FAP-α), thereby differing from that of normal fibroblasts that exhibit a CD34 immunophenotype [10,11].

The purpose of this study is to screen the expression of FAP-α around minuscule foci of microinvasion and investigate its functional role in the immunohistochemistic diagnosis of DCIS-MI.

## Methods

We used the surgical oncology breast cancer database to retrospectively evaluate 349 patients who had undergone mastectomy between March 1994 and March 2010. Archival material was obtained from the files of the Department of Pathology and Medical Records Room, the Forth Affiliated Hospital of Jinan University, Guangzhou, China. All specimens were formalin-fixed and paraffin-embedded. All the available slides were reviewed blindly by five pathologists simultaneously and a consensus report of the diagnosis was obtained. To test the validity of immunostains and for comparative evaluation, study cases were divided into 5 groups: group 1: normal mammary tissues from healthy women after plastic surgery, 20 cases; group 2: usual ductal hyperplasia (UDH), 72 cases; group 3: DCIS without microinvasion on H& E stain, 109 cases; group 4: DCIS with microinvasion on H & E stain (DCIS-MI) utilizing the AJCC criteria, 81 cases; group 5: invasive ductal carcinoma (IDC), 67 cases. This study was approved by our hospital review board.

Four sections of 4μ thickness were cut from the paraffin blocks of those breast lesions. Consecutive sections were used for a better comparison between morphology and protein expression. Immunohistochemical staining for CD34, smooth muscle actin (SMA), Calponin, and FAP-a was done as described elsewhere [12]. The primary antibodies against FAP-α (clone 427819, 1:50 dilution) and SMA (clone 1A4, 1:100 dilution) were obtained from R & D systems. The primary antibodies against Calponin (clone CALP, 1:50 dilution) was obtained from Abcam company, while that for CD34 (clone QBEnd10, 1:100 dilution) was obtained from Dako Corporation. Appropriate positive and negative controls were run in each case. In group 1, group 4, and group 5, staining was interpreted as positive only when cytoplasm staining was detected in more than 10% of stromal firoblasts in the microenvironment of the tumor-host interface. In group 2 and 3, staining was interpreted as positive only when more than 10% of stromal firoblasts cells in the microenvironment of the tumor-host interface or at the invasive front showed cytoplasm staining. Positive staining for Calponin in the myoepithelial cells (MECs) of breast duct was interpreted as continuously, discontinuously, scattered positive or negative.

## Results

Distinct immunohistochemical staining of CD34, SMA, Calponin and FAP-a was detected in all the 349 cases of this study. The association of the immunophenotype of stromal fibroblasts with various clinicopathological parameters is listed in Table 1.

**Table 1:** Levels of markers' expression in relation to clinicopathologic variables

Diagnostic group	Total cases	CD34		SMA		Calponin	FAP-α	
					
		Positive	%	Positive	%		Positive	%
Group 1	20	19	95.00	2	10.00	continuous (20)	0	0
Group 2	72	68	94.44	11	15.27	continuous (72)	1	1.39
Group 3	109	13	11.93	81	74.31	continuous (87) discontinuous (3) negative (19)	21	19.27
Group 4	81	16	19.75	77	95.06	continuous (0) discontinuously (17) scattered (3) negative (64)	67	82.72
Group 5	67	7	10.45	63	94.03	scattered (11) negative (56)	67	100

**Figure 1:**
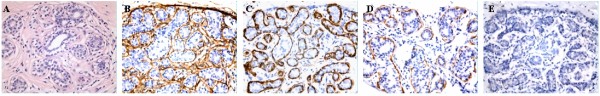
**Representative immunohistochemical staining results (magnification × 100)**. (a) HE; (b) CD34 positive; (c) SMA negative; (d) Calponin continuously positive; (e) FAP-a negative. The virtual slide for this article can be found here: http://www.diagnosticpathology.diagnomx.eu/vs/7086274655925365/1.

Group 1 (normal mammary tissues): As shown in Figure 1, CD34 protein expression was detected mainly in the cytoplasm of stromal fibroblasts in 18 (90%) of 20 cases. SMA protein expression was detected mainly in the cytoplasm of stromal fibroblasts in 4 (20%) of the total 20 cases. None of the 20 cases in this group showed positivity for FAP-a in the stroma. Calponin protein expression was detected continuously in the cytoplasm of MECs of breast duct in all the 20 cases.

**Figure 2:**
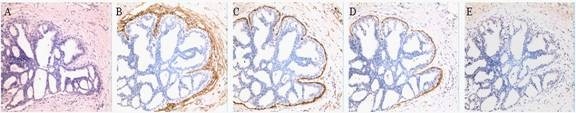
**Representative immunohistochemical staining results (magnification × 100)**. (a) HE; (b) CD34 positive; (c) SMA negative; (d) Calponin continuously positive; (e) FAP-a negative. The virtual slide for this article can be found here: http://www.diagnosticpathology.diagnomx.eu/vs/7086274655925365/2.

Group 2 (usual ductal hyperplasia, UDH): As shown in Figure 2, CD34 protein expression was detected mainly in the cytoplasm of stromal fibroblasts in 68 (94.44%) of the 72 cases. SMA protein expression was detected mainly in the cytoplasm of stromal fibroblasts in 11 (15.28%) of 72 cases. But only one (1.39%) of the 72 cases showed positivity for FAP-a in the stromal fibroblasts. Calponin protein expression was detected continuously in the cytoplasm of MECs of breast duct in all the cases.

**Figure 3:**
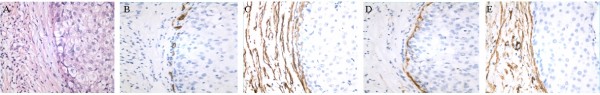
**Representative immunohistochemical staining results (magnification × 200)**. (a) HE; (b) CD34 negative; (c) SMA positive; (d) Calponin continuously positive; (e) FAP-a positive. The virtual slide for this article can be found here: http://www.diagnosticpathology.diagnomx.eu/vs/7086274655925365/3.

Group 3 (DCIS without microinvasion on H & E stain): As shown in Figure 3, CD34 protein expression was detected mainly in the cytoplasm of stromal fibroblasts in 13 (11.93%) of the total 109 cases. SMA protein expression was detected mainly in the cytoplasm of stromal fibroblasts in 81 (74.31%) of 109 cases. 21 (19.27%) of 109 cases showed fibroblasts that were FAP-a positive in the microenvironment of the tumor-host interface. Calponin protein expressed continuously in the cytoplasm of MECs of breast duct in 87 (79.82%) of 109 cases, whereas 3 (2.75%) cases showed scattered expression of Calponin. On the other hand, 19 (14.73%) cases showed Calponin negative. It was worth noting that of the 19 cases, especially in the region of the tumor-host interface where Calponin was negatively expressed, 19 (100%) cases showed FAP-a positive and 12 (63.16%) showed SMA positive, but 17 (89.47%) of these 19 cases reported negative for CD34 protein. Reexamination of these 19 cases was done blindly by the five pathologists using the technique of H & E and immunohistochemical staining. This led to the pathologists to change the diagnosis of 18 of these 19 cases to DCIS-MI.

**Figure 4:**
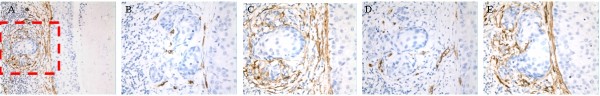
**Representative immunohistochemical staining results (magnification × 200)**. (a) HE; (b) CD34 negative; (c) SMA positive; (d) Calponin negative; (e) FAP-a positive. The virtual slide for this article can be found here: http://www.diagnosticpathology.diagnomx.eu/vs/7086274655925365/4.

Group 4 (DCIS with microinvasion on H & E stain, DCIS-MI): As shown in Figure 4, stromal fibroblasts in the tumor-host interface at the invasive front of DCIS-MI lesions were diagnosed. 16 (19.75%) of 81 cases showed CD34 positivity, whereas 77 (95.06%) of 81 cases showed SMA positivity, and 67 (82.72%) of 81 cases showed significant FAP-a positivity. In the tumor-host interface at the invasion front of all the 81 cases, 64 (79.01%) showed Calponin negative, 3 (3.70%) showed Calponin expression as scattered, and 17 (20.99%) showed Calponin discontinuously expressed in the crush artifactary and inflammatory remodeled stroma. Therefore, reexamination of the 17 cases was done on the basis of H & E and immunohistochemical staining. This led to the pathologists change the diagnosis of 13 of these 17 cases to DCIS without microinvasion.

**Figure 5:**
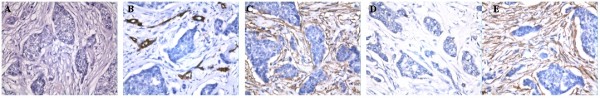
**Representative immunohistochemical staining results (magnification × 200)**. (a) HE; b CD34 negative; (c) SMA positive; (d) Calponin negative; (e) FAP-a positive. The virtual slide for this article can be found here: http://www.diagnosticpathology.diagnomx.eu/vs/7086274655925365/5.

Group 5 (invasive ductal carcinoma, IDC): As shown in Figure 5, stromal fibroblasts of 7 (10.45%) of 67 IDC cases showed CD34 positivity, while 63(94.03%) of 67 IDC cases showed significant SMA positivity. Moreover, all cases of IDC showed intense FAP-α staining of stromal fibroblasts. Calponin protein expression was detected sporadically positive or negative in the stroma of all the IDC cases.

## Discussion

DCIS-MI is a rare histological subtype of breast carcinoma. The cells deemed to be invasive must be distributed either singly or as small groups in a non-organoid pattern having irregular shapes that are reminiscent of the conventional invasive carcinoma with no particular orientation [4]. In the fourth edition of the *European guidelines for quality assurance in breast cancer screening and diagnosis *published in 2006 [13] and in the 2010 edition of the American Joint Committee on Cancer staging system [14], the diagnosis of microinvasive carcinoma of the breast (T1mic) is applied to those invasive carcinomas with no focus measuring > 1 mm. The current staging manual states that microinvasive carcinoma is nearly always encountered in a setting of DCIS [or, less often, lobular carcinoma in situ (LCIS)] in which small foci of tumor cells have invaded through the basement membrane into the surrounding stroma [15]. Although some authors have required that such invasive foci extend beyond terminal ductal lobular unit (TDLU) stroma into the interlobular tissue [16], this is not supported by the ultrastructural confirmation of intralobular capillaries in close proximity to the delimiting fibroblastic layer of terminal ducts [17,18]. Furthermore, DCIS often unfolds the TDLU externalizing the once intralobular stroma. Any distinctive feature of the TDLU stroma is often masked by an inflammatory infiltrate and/or stromal fibrosis. In the presence of DCIS, the TDLU stroma may not be distinguishable from the interlobular stroma [19].

In this study, all the cases were classified within the context of a study and were classified by the same pathologists repeatedly. Pathologists made the pathological diagnosis mainly based on morphological characteristics and the immunohistochemical staining according to that diagnostic criteria and the pathological diagnosis conclusion was unaffected by the archived diagnoses which had already reported to the clinician. In order to minimize the diagnostic subjectivity, we asked five experienced pathologists who had received the same training and mastered the diagnostic criteria, to review all the available slides and asked them to classify double-blindly. If their diagnoses are inconsistent, they would discuss together and make the final diagnosis. In this way, we kept our diagnostic results reliable.

But various patterns in DCIS may be mis-interpreted as stromal invasion, including the cases where there is degenerative appearance of the dislodged tumour cells, chronic inflammatory reaction, crush artefacts, cautery effects, and distortion or entrapment of involved ducts or acini by fibrosis [6-8,20]. As reported earlier [4], it is difficult to determine whether in-situ breast carcinoma is associated with microinvasion, even with the help of immunohistochemistry techniques.

The absence of basement membrane material around nests of tumor cells defines the process as being invasive. Immunohistochemistry for basement membrane components (laminin and type IV collagen) is helpful in detecting the presence or absence of basement membrane. However, cells of invasive cancer can still synthesize components of basement membrane around invasive nests. Therefore, the use of basement membrane markers for the detection of stromal invasion is not recommended [4,21].

The presence of MECs around nests of carcinoma cells defines the process as being in situ. Immunohistochemistry for P63, S100 and smooth-muscle myosin heavy chain (SMM-HC) for MECs has been used in determining whether a process represents in situ carcinoma or stromal invasion. But, those antibodies occasionally form an apparently discontinuous myoepithelial layer around nests of in situ lesions, and they also react with a small but significant subset of breast carcinoma tumor cells [4,21,22]. While MECs are retained around ductal-lobular spaces containing DCIS, molecular studies have indicated that MECs surrounding with mammary ducts and lobular acini have important roles in the development and physiology of normal mammary glands, including maintenance of the basement membrane around ductal-lobular structures, providing a physical barrier between epithelial cells and the surrounding stroma, and maintaining epithelial cell polarity. Furthermore, experimental evidence has indicated that MECs produce factors that, through paracrine effects, to inhibit tumor growth, invasion, and angiogenesis [23-26]. Recently, more and more attention has been paid to the potential role of the MECs in the progression of DCIS to invasive breast carcinoma. Though MECs that surround spaces involved by DCIS differ substantially from normal MECs in several respects [25-29], Calponin is a contractile element that expressed in differentiated smooth muscle cells and is highly sensitive to normal noninvasive MECs and breast MECs [30].

In this study, our diagnosis mainly based on morphological characteristics, while refering the expression of SMA, CD34 and FAP-α of stromal fibroblasts besides integrity of MECs. CD34 is a transmembrane glycoprotein expressed by haematopoietic stem cells, endothelial cells and mesenchymal cells in different tissues including breast that is thought to be involved in the modulation of cell adhesion and signal transduction. CD34^+ ^fibrocytes/fibroblasts derive from myeloid precursors, besides its function as a matrix-production cell, it is a potent antigen-presenting cell and therefore it has been claimed that CD34^+ ^may play a role in host response to tissue damage [31-33]. Different studies have shown that the presence or absence of this population of cells might be useful in distinguishing benign from malignant lesions of the skin [34] and gastrointestinal tract [35,36]. The stroma of normal mammary gland contains many CD34 positive fibroblasts/fibrocytes and the presence of stromal positive CD34 fibroblasts has been shown to be associated with benign lesions [31,32,37]. In malignant tumors it was noticed a loss of CD34 positive cells and gain of smooth muscle cell actin positive myofibroblasts [33,38,39] and this is in keeping with our findings. In our study, stromal fibroblasts in the normal mammary tissues and UDH showed mainly immunophenotype of SMA^-^CD34^+^FAP-α^-^. In the tumor-host interface of DCIS and IDC, stromal fibroblasts exhibited mainly immunophenotype of SMA^+^CD34^-^FAP-α^+^. In the tumor-host interface of DCIS-MI, stromal fibroblasts exhibited SMA^-^CD34^+^FAP-α^-^, however, in the tumor-host interface at the invasive front of DCIS-MI lesions, stromal fibroblasts exhibited mainly immunophenotype of SMA^+^CD34^-^FAP-α^+^.

To the best of our knowledge, there is no study evaluating the role of FAP-α with reference to microinvasion of DCIS.

FAP-α is a cell surface glycoprotein belonging to the serine protease family, is expressed by the CAFs in over 90% of human epithelial cancers including breast, ovarian, bladder, colorectal, and lung cancers, but it is not expressed in epithelial cancer cells, normal fibroblasts, and other normal tissues except the transient expression in healing wounds [40-45]. In this study, at the invasion front of all the DCIS-MI, stromal fibroblasts expressed FAP-α and there was significant statistical difference in the expression of FAP-α protein in groups 3 and 4, but no statistical difference was reported in the FAP-α protein expression for groups 4 and 5. FAP-α plays an important role in tumor growth and metastasis, as its expression on CAFs may create an environment permissive for cancer growth and invasion via collagenase and dipeptidyl peptidase activities [10,11,46,47]. We suggested the possibility of FAP-α promoting the formation of microemboli that facilitates the metastasis of breast cancer. Thus, FAP-α can serve as a novel marker for pathologically determining whether DCIS has microinvasion.

Based on immunohistochemical staining, we found that some initally diagnosed DCIS-MI had discontinuously MECs around the so-called invasive foci without stromal reaction (SMA^-^CD34^+^FAP-α^-^). In such condition, we changed the original diagnosis of DCIS-MI to DCIS. In the same way, some of the DCIS cases were rediagnosed as DCIS-MI. In the study group 3, at the invasive front of the 18 re-examined DCIS-MI, 12 cases showed SMA positive, while 5 cases showed CD34 positive. 17 cases showed Calponin negative, and one Calponin negative case was still diagnosed as DCIS, but all of the 18 re-examined cases showed negative expression of FAP-α in the stromal fibroblasts. The sensitivity of SMA, CD34, Calponin and FAP-α in diagnosis of DCIS is 75%, 78%, 98% and 98%, respectively. In the study group 4, in the tumor-host interface of the 13 re-examined DCIS cases, 8 cases showed SMA positive, while 7 cases showed CD34 negative. 4 cases showed Calponin negative, and 12 cases showed FAP-α negative. The sensitivity of SMA, CD34, Calponin and FAP-α in diagnosis of DCIS-MI is 77%, 71%, 97% and 98%, respectively. In our study, using Calponin and FAP-α adjunctively improved the sensitivity of pathological diagnosis of DCIS by 13.6%, whereas the adjunctive use of Calponin and FAP-α improved the sensitivity of pathological diagnosis of DCIS-MI by 11.29%.

In conclusion, this study provides the first evidence that immunostaining with FAP-α and Calponin can serve as a novel marker for pathologically diagnosing whether DCIS has microinvasion. We also suggested the possibility of FAP-α promoting the formation of microemboli, which facilitate the metastasis of breast cancer.

## Competing interests

The authors declare that they have no competing interests.

## Authors' contributions

XH and LNY participated in the design of the study and wrote the manuscript. XXH, ZXL and QX carried out the H&E and IHC staining. XH and LNY collected the clinical data and reviewed H&E and IHC slides. All authors read and approved the final manuscript.
